# The influence of sleep duration on patients with coronary artery disease: a four-year observational study

**DOI:** 10.3389/fendo.2025.1555880

**Published:** 2025-05-05

**Authors:** Runfeng Ma, Ruoyu Wang, Bingqian Wang, Zihan Tang, Tian Qiu, Yiduo Lu, Gang Liu

**Affiliations:** ^1^ International Medical College, Chongqing Medical University, Chongqing, China; ^2^ College of Basic Medical Sciences, Naval Medical University, Shanghai, China; ^3^ Department of Cardiovascular Medicine, Cardiovascular Research Center, The First Affiliated Hospital of Chongqing Medical University, Chongqing, China

**Keywords:** sleep duration, coronary artery disease, CAD-related mortality, myocardial infarction, Southwest China

## Abstract

**Background:**

Coronary artery disease (CAD) is highly prevalent and fatal worldwide. In China, particularly in the southwest region, the association between CAD and sleep duration remains insufficiently understood. This study aims to investigate outcomes among CAD patients with varying sleep duration.

**Methods:**

In a Southwest Chinese cohort, patients with CAD were categorized into three sleep duration groups: <6 hours, 6–8 hours, and >8 hours. Over a four-year follow-up, the endpoint including new-onset myocardial infarction and CAD-related deaths was recorded. The Fine-Gray model was employed to evaluate the estimated marginal occurrence probability of the target event. Additionally, Kaplan-Meier estimation and Cox regression analysis were conducted to further investigate the association between sleep duration and outcomes.

**Results:**

The study enrolled 816 residents with CAD, who had an average age of 69.2 ± 8.3 years old, of whom 40.2% were male. Across the three sleep duration groups (6-8h, <6h, and >8h), the hazard ratios (HRs) with 95% confidence interval for new-onset myocardial infarction were: 1.00 (reference), 2.67 (1.57-4.55) (*P* < 0.001), and 0.98 (0.30-3.21) (*P*=0.970). For CAD-related mortality, the HRs were: 1.00 (reference), 5.20 (2.53-10.68) (*P* < 0.001), and 5.02 (1.59-15.80) (*P*=0.006). This trend was consistently observed in both the Fine-Gray model and subgroup analyses.

**Conclusions:**

Both short (<6 hours/day) and long (>8 hours/day) sleep duration were linked to an elevated risk of cardiac mortality among CAD patients in Southwest China. Short sleep duration was also found to be associated with high myocardial infarction risk.

## Introduction

Coronary artery disease (CAD) is one of the leading causes of morbidity and mortality worldwide, placing a substantial economic burden on healthcare systems (1, 2). Numerous risk factors have been associated with the incidence and prevalence of CAD, including hypertension, diabetes mellitus (DM), smoking, hyperlipidemia, and physical inactivity (3). However, the relationship between sleep duration and CAD remains inconclusive.

Recently, sleep habits have gained increasing attention in cardiovascular research due to their potential link with adverse health outcomes (4–7). Several studies have suggested that insufficient sleep duration is associated with a higher risk of developing CAD (8, 9). However, findings have varied across different geographic regions, with some studies reporting contradictory results in diverse populations (10, 11). Moreover, prior research has primarily focused on the general healthy population rather than on individuals diagnosed with CAD.

There remains a significant gap in research examining the association between sleep duration and CAD-related mortality, as well as the incidence of new-onset myocardial infarction (MI) in China. Therefore, this study aims to provide further insights into these associations within this specific demographic.

## Methods

### Study design

This study is a sub-project of China’s National Key Research and Development Program (2018YFC1311400), focusing on a population from Chongqing, a major city in Southwest China. From 2018 to 2020, under the supervision of the Health Commission and the Center for Disease Control and Prevention, a cross-sectional stratified random sampling survey was conducted among residents in 16 large communities by medical staff from local medical institutions. Data collection included medical record reviews, face-to-face questionnaire surveys, and occasional telephone interviews, ensuring comprehensive baseline characteristics and disease information. Physical examinations and serum biochemical tests were conducted as the final medical consultation with healthcare professionals.

**Figure 1:**
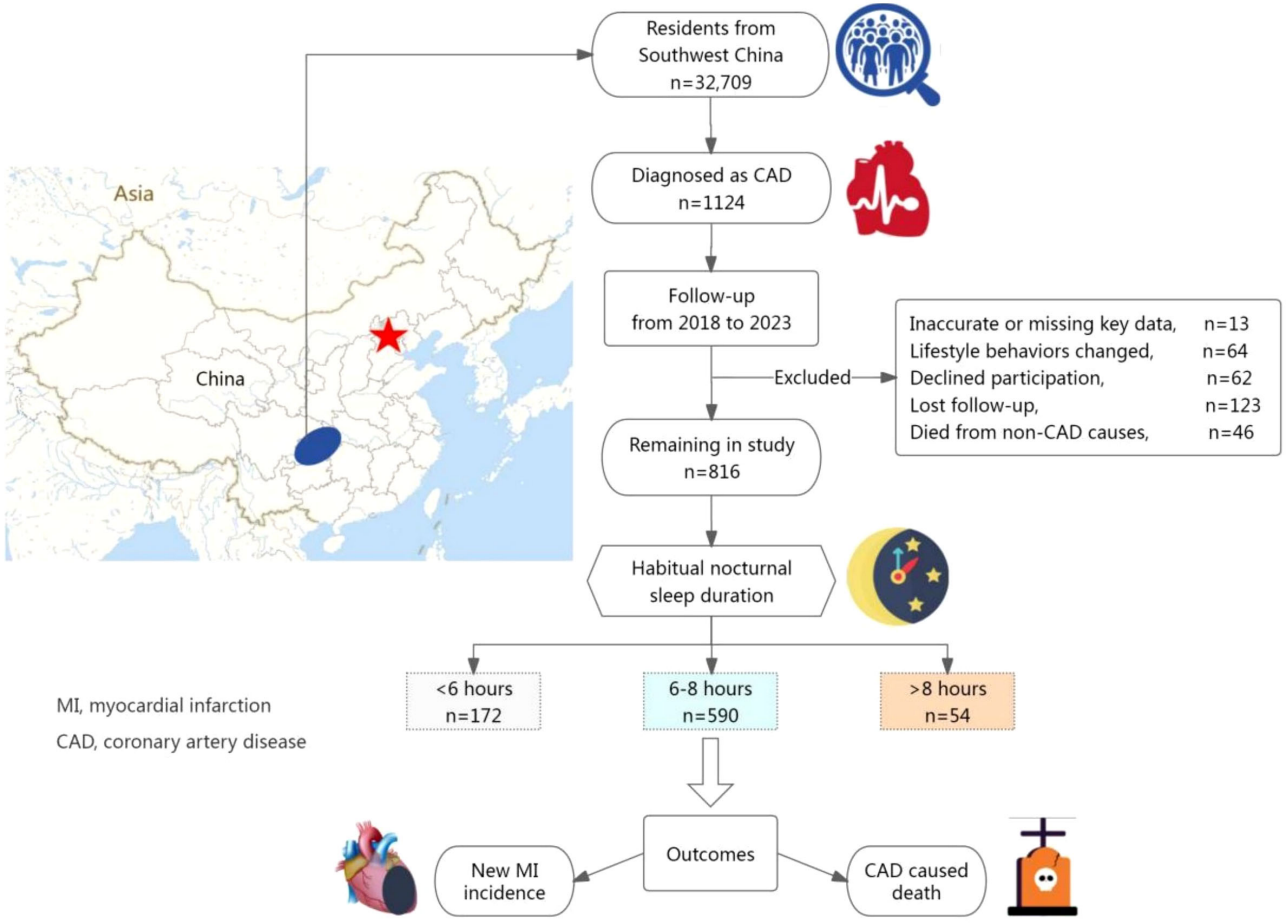
Flowchart of the study design.

Of the 32,709 participants initially enrolled (aged ≥18 years), 1,124 had a confirmed diagnosis of CAD at baseline. Thirteen participants were excluded due to missing or inaccurate key data. Between 2018 and 2023, participants underwent follow-ups every six months, primarily via telephone or face-to-face interviews, supplemented by medical record reviews. During this period, 64 patients were excluded due to changes in sleep habits (n=19) or other significant lifestyle modifications (n=45). Additionally, 62 participants declined follow-up, 46 died from non-CAD causes, and 123 were lost to follow-up. Ultimately, 816 CAD patients were included in the final analysis (see **Figure 1**).

Participants were categorized into three groups based on their sleep duration: <6 hours, 6–8 hours, and >8 hours. All medical information was verified through evidence-based medical records. The primary study endpoints were new-onset MI and CAD-related mortality.

This study was led by the Department of Cardiovascular Medicine at the First Affiliated Hospital of Chongqing Medical University and was approved by its Ethics Committee (No.2020-233). Informed consent was obtained from all participants.

### Definition of terms

In this study, sleep duration is defined as an individual’s consistent total habitual nighttime sleep per day over the past five years (12, 13). Participants reported their habitual bedtime, wake-up time, the frequency of nocturnal awakenings, and the average duration of each awakening, which were used to calculate sleep duration. A small subset of participants utilized electronic devices (e.g., smartwatches) to assist in recording sleep patterns. Changes in sleep habits are characterized as alterations in an individual’s average daily sleep time exceeding two hours, sustained for more than three months (14).

Cardiovascular disease (CVD) family history refers to an individual with a medically documented first-degree relative who has been diagnosed with CVD. The term “well-educated” applies to residents who have completed high school or attained higher education. “High income” is defined as an average monthly income exceeding 5,000 yuan.

Habitual lifestyle behaviors were retrospectively assessed over a five-year period prior to enrollment. The term “solitary living” indicates that the individual spends an average of 26 or more days per month living independently. “Smoking” and “alcohol consumption” are defined as engaging in these activities at least three times per week. “Physical inactivity” is classified as participating in less than 20 hours of outdoor physical labor or exercise weekly (13). A high-salt or high-fat diet refers to a dietary preference for foods rich in salt (e.g., pickled and preserved items) or high in fat (e.g., fried foods and fatty meats). Conversely, the phrase “favoring vegetables, fruits, and yogurt” signifies consuming these food groups at least five days per week. Participants who exhibited continuous changes in habitual lifestyle behaviors for more than three months during the follow-up were excluded.

Diagnosis required medical examination or documentation from secondary or tertiary healthcare institutions. CAD diagnosis was based on percutaneous coronary angiography or computed tomography angiography confirming significant narrowing, defined as ≥50% stenosis in at least one coronary artery. MI diagnosis was established when serum markers indicative of cardiac injury were elevated, in conjunction with at least one of the following criteria: (a) Myocardial ischemic symptoms; (b) New electrocardiogram (ECG) changes; (c) Imaging evidence of reduced viable myocardium or abnormal wall motion patterns. CAD-related mortality refers specifically to deaths medically recorded as resulting from CAD or its complications, excluding fatalities due to other diseases or accidents.

### Data analysis

Continuous variables were expressed as mean ± standard deviation (SD) for normally distributed data. Analysis of Variance (ANOVA) was applied to assess differences among groups when the assumption of homogeneity of variance was met. For data that did not meet this assumption, values were reported as median and interquartile range (IQR, 25%–75%), and the Kruskal-Wallis test was used for group comparisons. Categorical variables were summarized as frequency and percentage (n%), with differences across groups assessed using Pearson’s *χ*² test.

To account for competing risks, 46 cases of non-CAD-related deaths were incorporated into the analysis, and the Fine-Gray model was applied to estimate the marginal occurrence probability of the target event. The Kaplan-Meier method was used to evaluate the association between sleep duration categories and outcome events. Univariate Cox regression analysis was conducted to identify associations between baseline characteristics and endpoint events. Variables that showed statistical significance (*P* < 0.05) were subsequently included in multivariate analyses to compute the hazard ratio (HR) with a 95% confidence interval (CI). To further validate the robustness of the findings, subgroup analyses were performed.

All statistical analysis was conducted using IBM SPSS Statistics version 28 (IBM Corporation, Armonk, NY, USA) and the R programming language (version 4.2.2). Graphical visualizations were generated using Prism version 9. A two-sided *P*-value < 0.05 was considered statistically significant for all tests.

## Results

**Figure 2:**
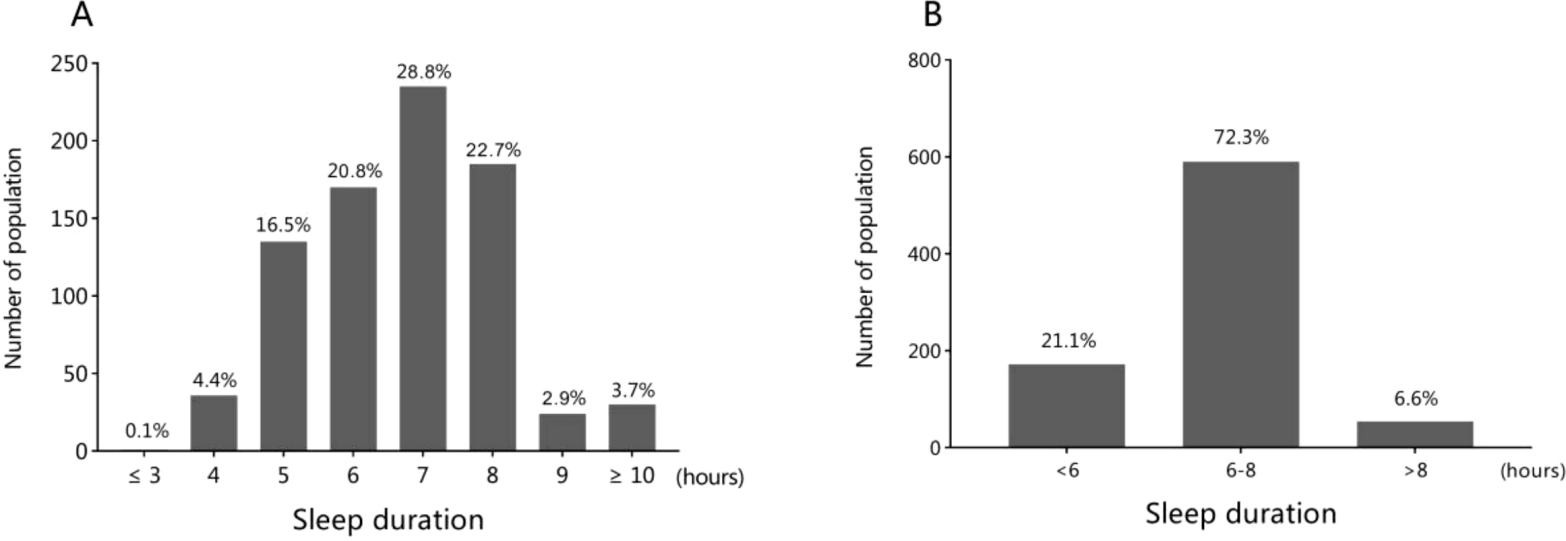
The distribution of sleep duration among the general population **(A)** and the three groups **(B)**.

The prevalence of CAD within this cohort was 3.4% (1,124 out of 32,709 individuals). A total of 816 CAD patients were included in the study, 40.2% of whom were male, with a mean age of 69.2 ± 8.3 years (range: 27.0–92.0 years) at enrollment. The distribution of daily sleep duration is presented in **Figure 2**, showing that most participants slept for 6 to 8 hours per day. During the four-year follow-up, a total of 66 cases (8.1%) of new-onset MI, and 40 cases (4.9%) of CAD-related death were recorded.

As shown in **Table 1**, CAD patients with a sleep duration of less than six hours tended to be older and had a higher likelihood of having a family history of CVD as well as living alone. They were also more likely to consume alcohol, engage in low levels of physical activity, follow a high-salt diet, and exhibit higher HDL-C levels. In contrast, CAD patients with a sleep duration of 6–8 hours tended to be better educated and had a greater preference for fruits and yogurt in their diet. Moreover, they had greater height and body weight. Apart from these findings, there were no significant differences among the three groups in terms of demographics, lifestyles, physical examinations, or serum biochemical test results.

**Table 1:** Demographic, clinical characteristics and outcomes of CAD patients among different groups.

	Overall	Sleeping duration, hrs/day			
Characteristics	(n=816)	< 6h (n=172)	6-8h (n=590)	> 8h (n=54)	*P value*
Demographics					
Enrollment age, yrs	69.21 ± 8.28	71.02 ± 6.48	68.74 ± 8.70	68.65 ± 7.97	0.005
Male gender, n%	328 (40.2)	63 (36.6)	243 (41.2)	22 (40.7)	0.560
CVD family history	108 (13.2)	45 (26.2)	61 (10.3)	(3.7)	< 0.001
Well educated, n%	119 (14.6)	8 (4.7)	103 (17.5)	8 (14.8)	< 0.001
High income, n%	57 (7.0)	5 (2.9)	47 (8.0)	5 (9.3)	0.058
Lifestyles					
Solitary living, n%	80 (9.8)	25 (14.5)	52 (8.8)	3 (5.6)	0.047
Smoking, n%					
Never, n%	700 (85.8)	146 (84.9)	508 (86.1)	46 (85.2)	0.269
Ever, n%	30 (3.7)	17 (2.9)	11 (6.4)	2 (2.3)	
Current, n%	86 (10.5)	65 (11.0)	15 (8.72)	6 (11.1)	
Alcohol consumption, n%					
Never, n%	723 (88.6)	148 (86.0)	522 (88.5)	53 (98.1)	0.040
Ever, n%	27 (3.3)	14 (2.4)	13 (7.6)	0 (0.0)	
Current, n%	66 (8.1)	54 (9.2)	11 (6.4)	1 (1.9)	
Physical inactivity, n%	260 (31.9)	66 (38.4)	184 (31.2)	10 (18.5)	0.019
High-salt diet, n%	171 (21.0)	54 (31.4)	109 (18.5)	8(14.8)	< 0.001
High-fat diet, n%	70 (8.6)	13 (7.6)	51 (8.6)	(11.1)	0.714
Vegetables diet, n%	787 (96.4)	167 (97.1)	566 (95.9)	54 (100.0)	0.265
Fruits diet, n%	379 (46.5)	49 (28.5)	304 (51.5)	26 (48.2)	< 0.001
Favor yogurt, n%	125 (15.3)	12 (7.0)	108 (18.3)	5 (9.3)	< 0.001
Physical examinations					
Height, cm	156.56 ± 8.79	154.07 ± 8.56	157.24 ± 8.78	157.13 ± 8.44	< 0.001
Weight, kg	61.32 ± 10.03	59.33 ± 9.73	61.99± 10.13	60.32 ± 9.04	0.007
BMI, kg/m2	25.03 ± 3.75	25.03 ± 3.91	25.08 ± 3.75	24.43 ± 3.16	0.479
Heart rate, bpm	74.23 ± 11.69	74.47 ± 13.39	74.08 ± 11.12	75.17 ± 12.24	0.774
SBP, mmHg	139.52 ± 15.87	140.27 ± 17.59	139.36 ± 15.60	138.91 ± 12.97	0.769
DBP, mmHg	81.50 ± 10.69	81.41 ± 10.75	81.36 ± 10.75	83.39 ± 9.88	0.406
Serum biochemical tests					
TG, mmol/L	1.39 (1.03-2.10)	1.38 (0.99-2.00)	1.40 (1.03-2.10)	1.35 (0.96-1.97)	0.306
TC, mmol/L	4.76 (4.06-5.48)	4.78 (4.10-5.62)	4.77 (4.05-5.45)	4.55 (3.92-5.26)	0.382
HDL-C, mmol/L	1.42 (1.17-1.71)	1.46 (1.21-1.71)	1.44 (1.17-1.75)	1.25 (1.08-1.43)	0.001
LDL-C, mmol/L	2.33 (1.89-3.00)	2.52 (1.94-3.00)	2.29 (1.87-3.04)	2.33 (1.96-2.78)	0.314
Fasting glucose, mmol/L	5.40(4.81-6.24)	5.21 (4.70-6.03)	5.42 (4.85-6.30)	5.22 (4.70-5.86)	0.052
Comorbidity at baseline					
Hypertension, n%	647 (79.3)	136 (79.1)	471(79.8)	40 (74.1)	0.605
Hyperlipidemia, n%	460 (56.4)	89 (51.7)	344 (58.3)	27 (50.0)	0.193
DM, n%	176 (21.6)	41 (23.8)	120 (20.3)	15 (27.8)	0.320
Obesity, n%	150 (18.4)	35 (20.3)	106 (18.0)	9 (16.7)	0.734
Stroke, n%	82 (10.0)	25 (14.5)	53 (9.0)	4(7.4)	0.083
Heat failure, n%	47 (5.8)	39 (22.7)	8 (1.4)	0(0.0)	< 0.001
Atrial fibrillation, n%	41 (5.0)	7 (4.1)	33 (5.6)	1(1.9)	0.393
PAD, n%	16 (2.0)	3 (1.7)	11 (1.9)	2(3.7)	0.492
Medications at baseline					
Anti-PLT, n%	98 (12.0)	20 (11.6)	72 (12.2)	6 (11.1)	0.958
Anticoagulants, n%	13 (1.6)	1 (0.6)	10 (1.7)	2(3.7)	0.219
Antihypertensive drugs, n%	513 (62.9)	108 (62.8)	376 (63.7)	29 (53.7)	0.345
Antiglycemic drugs, n%	144 (17.6)	33 (19.2)	101 (17.1)	10 (18.5)	0.810
Lipid-lowering drugs, n%	136 (16.7)	25 (14.5)	106 (18.0)	5 (9.3)	0.181
Medications at follow-up					
Anti-PLT n%	183 (22.4)	33 (19.2)	137 (23.2)	13 (24.1)	0.513
Anticoagulants, n%	20 (2.5)	2 (1.2)	16 (2.7)	2(3.7)	0.364
Antihypertensive drugs, n%	537 (65.8)	111 (64.5)	393 (66.6)	33 (61.1)	0.663
Antiglycemic drugs, n%	161 (19.7)	35 (20.4)	115 (19.5)	11 (20.4)	0.962
Lipid-lowering drugs, n%	191 (23.4)	36 (20.9)	148 (25.1)	7 (13.0)	0.091
Endpoints					
New MI, n%	66 (8.1)	25 (14.5)	38 (6.4)	3 (5.6)	0.002
CAD caused Death, n%	40 (4.9)	22 (12.8)	14 (2.4)	4(7.4)	< 0.001

CVD, cardiovascular disease; BMI, body mass index; SBP, Systolic blood pressure; DBP, diastolic blood pressure; TG, triglycerides; TC, total cholesterol; HDL-C, high density lipoprotein cholesterol; LDL-C, low density lipoprotein cholesterol; DM, diabetes mellitus; PAD, peripheral arterial disease; PLT, platelet; MI, myocardial infarction; CAD, coronary artery disease.

The overall prevalence of comorbid conditions was as follows: obesity (18.4%), hypertension (79.3%), DM (21.6%), hyperlipidemia (56.4%), stroke (10.0%), heart failure (5.8%), atrial fibrillation (5.0%), and peripheral artery disease (2.0%). However, at follow-up, individuals with less than 6 hours of sleep had the highest rates of new-onset MI (14.5%) and CAD-related mortality (12.8%).

**Figure 3:**
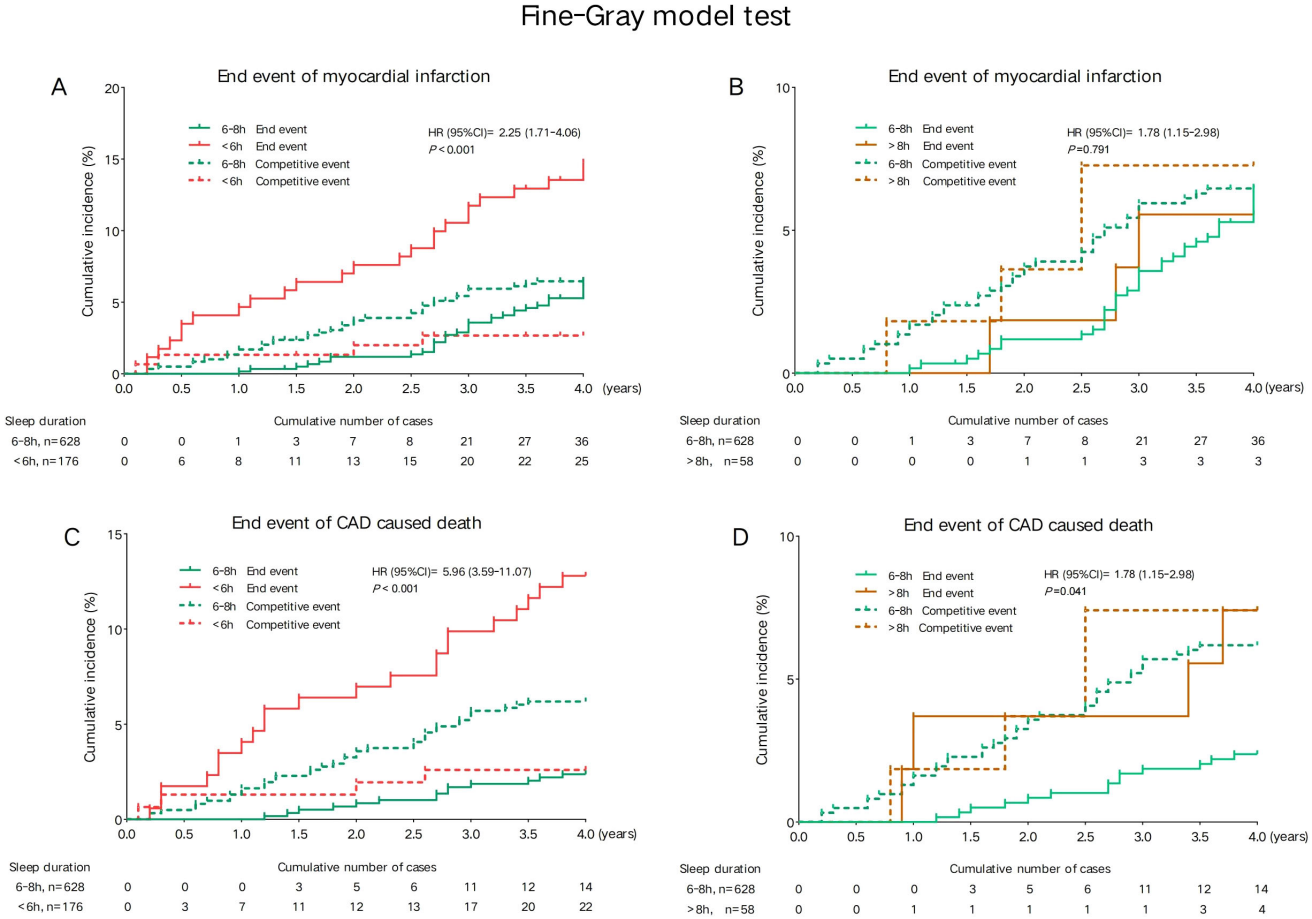
Univariate competing risk analysis model for endpoint events among sleep duration groups. **(A)** Cumulative incidence of myocardial infarction in participants with sleep duration <6 h versus 6-8 h. **(B)** Cumulative incidence of myocardial infarction in participants with sleep duration >8 h versus 6-8 h. **(C)** Cumulative incidence of CAD-related death in participants with sleep duration <6 h versus 6-8 h. **(D)** Cumulative incidence of CAD-related death in participants with sleep duration >8 h versus 6-8 h. Cumulative incidence curves were generated using the Fine-Gray competing risk model. Solid lines represent the incidence of the target end event (myocardial infarction or CAD-related death), while dashed lines represent competing events. Hazard ratios (HRs) with 95% confidence intervals (CIs) and corresponding *P*-values are provided within each panel.

In the Fine-Gray model, after accounting for competing events in the univariate proportional hazards analysis, a sleep duration of less than 6 hours was associated with a higher risk of MI (HR 2.25, 95% CI 1.71-4.06, *P* < 0.001) and CAD-associated mortality (HR 5.96, 95% CI 3.59-11.07, *P* < 0.001). Additionally, a sleep duration exceeding 8 hours was also linked to an increased risk of CAD-related death (HR 1.78, 95% CI 1.15-2.98, *P* < 0.001) (refer to **Figure 3**).

**Figure 4:**
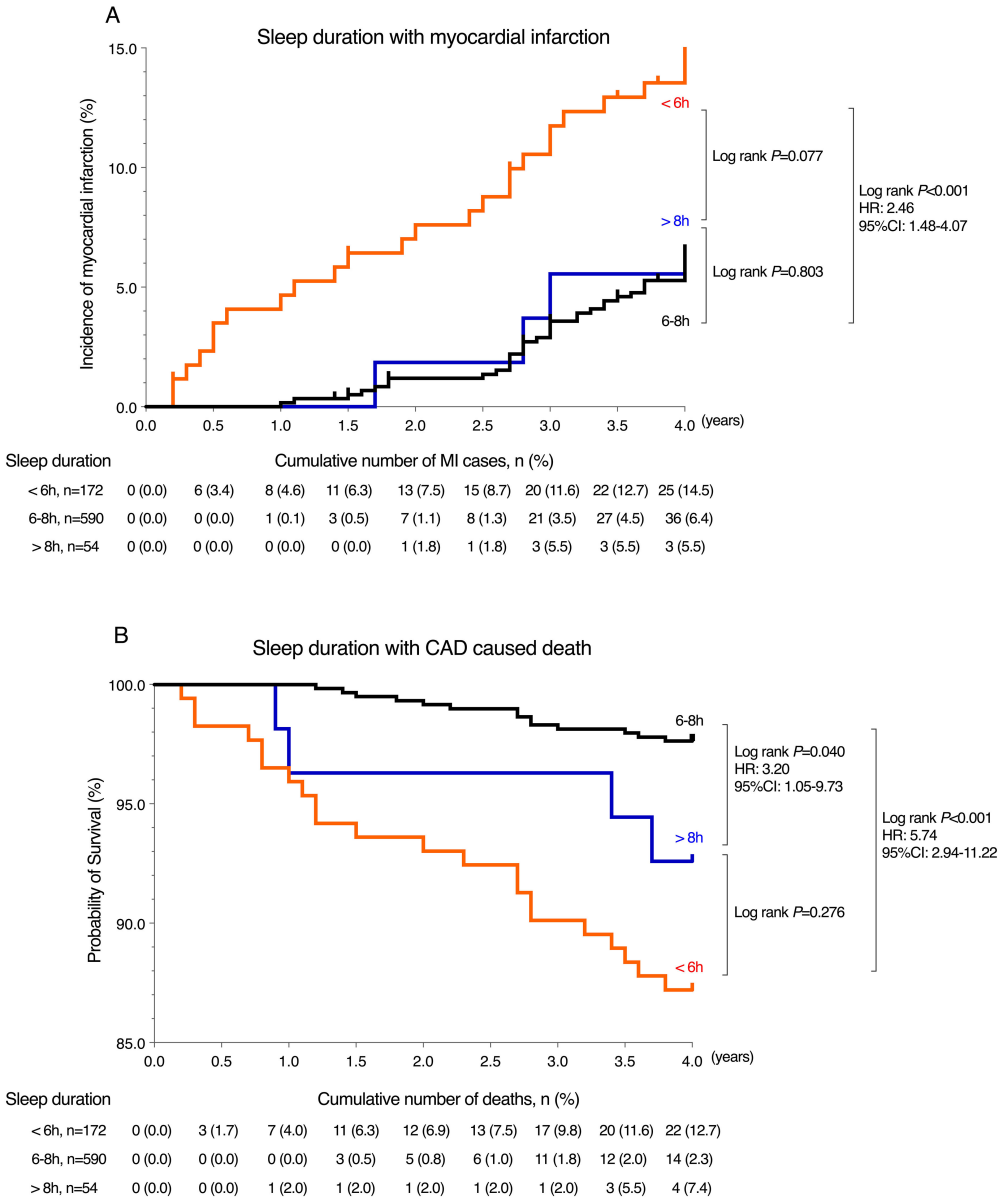
Kaplan-Meier analysis for endpoint events among sleep duration groups. **(A)** Incidence of myocardial infarction by sleep duration (<6 h, 6-8 h, >8 h). Short sleep (<6 h) was associated with significantly higher risk compared to 6-8 h (HR=2.46, 95% CI: 1.48-4.07, *P* < 0.001). No significant difference was found for >8 h (*P*=0.803). **(B)** Survival probability for CAD-related death by sleep duration. Short sleep (<6 h) showed the highest mortality risk (HR = 5.74, 95% CI: 2.94-11.22, *P* < 0.001), followed by >8 h (HR=3.20, 95% CI: 1.05-9.73, *P*=0.040). The black curve represents the group with 6-8 hours sleep, red curve represents the group with less than 6 hours sleep, and blue curve represents the group with more than 8 hours sleep.

Kaplan-Meier (KM) curves indicated that compared to the 6–8 hour sleep duration group, those with less than 6 hours of sleep had the highest risk of developing new MI, with an HR of 2.46 (95% CI: 1.48-4.07) (log-rank *P* < 0.001). Both short sleep duration (<6h) and long sleep duration (>8h) increased the risk of CAD-related mortality, with HRs of 5.74 (95% CI 2.94–11.22, log-rank *P* < 0.001) and 3.20 (95% CI 1.05–9.73, log-rank *P* = 0.040), respectively (refer to **Figure 4**).

**Figure 5:**
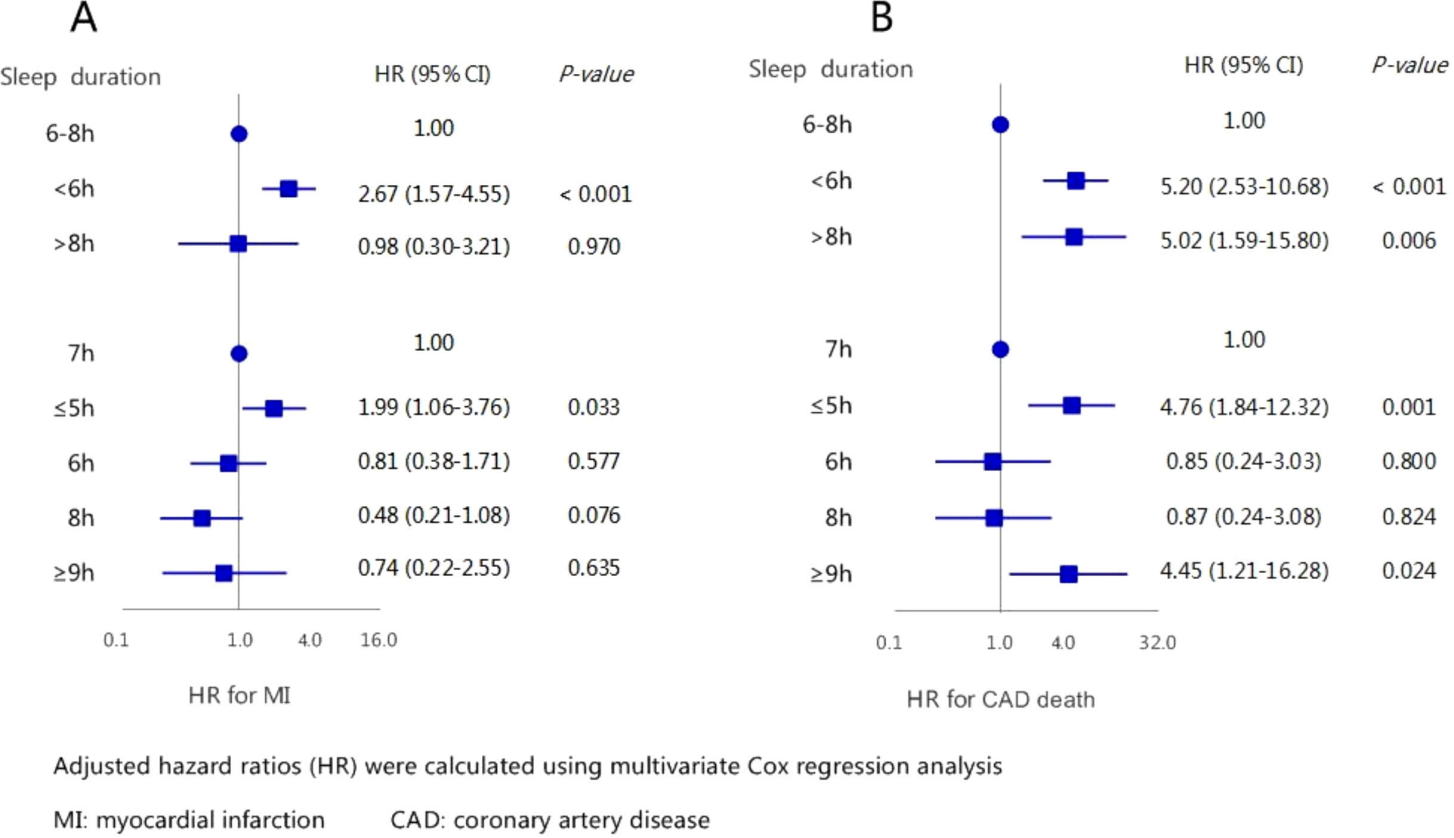
The association between sleep duration with new-onset myocardial infarction **(A)** and CAD caused death **(B)** by multivariate analysis.

As displayed in **Figure 5**, in the multivariate Cox regression model, the HR (95% CI) for new-onset MI across the three sleep duration groups (6-8h, <6h, and >8h) were: 1.00, 2.67 (1.57-4.55) (*P* < 0.001), and 0.98 (0.30-3.21) (*P*=0.970). Among the five sleep duration groups (7h, <5h, 6h, 8h and >9h) were: 1.00, 1.99 (1.06-3.76) (*P*=0.033), 0.81 (0.38-1.71) (*P*=0.577), 0.48 (0.21-1.08) (*P*=0.076), and 0.74 (0.22-2.55) (*P*=0.635), respectively. For CAD-related mortality, the HR (95% CI) across the three sleep duration groups (6-8h, <6h, and >8h) were: 1.00, 5.20 (2.53-10.68) (*P* < 0.001), and 5.02 (1.59-15.80) (*P*=0.006). Among the five sleep duration groups (7h, <5h, 6h, 8h and >9h) were: 1.00, 4.76 (1.84-12.32) (*P*=0.001), 0.85 (0.24-3.03) (*P*=0.800), 0.87 (0.24-3.08) (*P*=0.824), and 4.45 (1.21-16.28) (*P*=0.024), respectively. The relationships between the remaining confounding factors and the endpoint events are presented in **Supplementary Tables 1**, **2**.

In the subsequent subgroup analysis, when participants were classified into short (<6h) versus referent (6-8h) sleep duration groups (**Supplementary Figures 1**, **3**), and long (>8h) versus referent (6-8h) sleep duration groups (**Supplementary Figures 2**, **4**), the findings in most subgroups remained largely consistent with those observed in the overall population.

## Discussion

This retrospective study exclusively included patients diagnosed with CAD in Southwest China and demonstrated that: (1) the prevalence of CAD in this population was 3.4%. Among these individuals, over a four-year follow-up, the overall incidence rate of new-onset MI was 8.1%, while the mortality rate caused by CAD was 4.9%; (2) insufficient sleep duration (<6h) was identified as an independent risk factor for both new-onset MI and CAD-related mortality; and (3) excessive sleep time (>8h) was also associated with an increased risk of CAD-related death.

### Prevalence of CAD and MI

CAD and MI remain major global public health concerns. Epidemiological data indicate that the prevalence of CAD ranges from approximately 5% to 8% worldwide (15), while the prevalence of MI has been reported at 3.8% among individuals younger than 60 years and 9.5% among those older than 60 years (16). The rising incidence of these conditions places a considerable economic and healthcare burden on society. A recent study by the American Heart Association estimated that approximately 15.4 million individuals over the age of 20 in the United States have ischemic heart disease (17), corresponding to an overall CAD prevalence of 6.4% in this population (7.9% in men and 5.1% in women). Additionally, the prevalence of MI in the United States has been estimated at 2.9% (4.2% in men and 2.1% in women) (17).

This trend is consistent in other developed countries but not in developing nations, where the number of deaths due to CAD is projected to rise. In India, CAD prevalence ranges from 2.5% to 12.6% in urban areas and 1.4% to 4.6% in rural areas (18). In China, the prevalence of CAD is around 3.2% (19) and in this large population from Southwest China was 3.4%. The prevalence rates vary with different sample sizes and regions. Compared to other regions, the prevalence in China is relatively low; however, due to its large population, China still faces a significant CAD crisis.

Furthermore, the prevalence of MI in China is estimated at 1.3% in urban areas and 1.6% in rural areas, reflecting an increase of 0.55% over 17 years (20, 21). In comparison, the United States reports approximately 0.79 million new MI cases annually, with an estimated MI-related mortality of up to 1 million per year (22, 23). These statistics underscore the substantial public health burden posed by MI in China, highlighting the need for enhanced preventive measures and healthcare interventions (24).

Our study observed a higher prevalence of CAD in Southwest China, which may be attributable to multiple environmental and geographic factors. High-altitude regions are associated with chronic hypoxia, which can lead to vascular constriction and elevated blood pressure, thereby increasing CAD risk (25). Additionally, urban areas in Southwest China experience higher levels of air pollution, particularly fine particulate matter (PM2.5) exposure, which has been linked to systemic inflammation and heightened cardiovascular risk (26).

Dietary habits in this region may also contribute to the increased CAD burden. The traditional preference for high-salt and high-fat diets raises the risk of cardiovascular events. Furthermore, disparities in healthcare accessibility across different regions may result in variations in CAD diagnosis and management. These regional differences highlight the need for tailored public health interventions and healthcare strategies to address CAD risk factors specific to Southwest China.

### Sleep duration with MI

The association between sleep duration and MI has been widely investigated, yet findings remain inconsistent. In the Chinese population, Lian et al. (27) identified sleep insufficiency as a significant risk factor for acute MI. In contrast, Ye et al. (28) reported no significant relationship between either short or long sleep duration and MI.

Our findings regarding short sleep duration are consistent with the majority of previous studies, suggesting that insufficient sleep is associated with a heightened risk of new-onset MI (29, 30). Specifically, our study demonstrated that patients who slept less than 6 hours per night had a significantly higher risk of MI compared to those with normal sleep duration (HR: 2.67, 95% CI: 1.57–4.55). Similarly, Daghlas et al. (31) and Lian et al. (27) reported that sleep duration of less than 6 hours was a significant contributor to acute MI, with an HR (95% CI) of 1.20 (1.07–1.33) and an Odds Ratio (OR) (95% CI) of 2.97 (1.95–4.52), respectively. However, variations in study populations, follow-up durations, and patient characteristics may account for discrepancies in findings, as some studies have not observed a significant association (28).

The evidence regarding long sleep duration is even more heterogeneous. Our study found no significant relationship between sleeping more than 8 hours per night and MI incidence, a finding consistent with the results of Wang et al. (32) and Ye et al. (28), who also reported no significant association between long sleep duration and MI risk. Ye et al. further noted that prolonged sleep duration was associated with an elevated risk of stroke but not MI. Conversely, other studies, such as that conducted by Daghlas et al. (31), reported a 34% increased risk of incident MI with prolonged sleep duration.

Several demographic and clinical factors may explain these discrepancies. For instance, Ye et al.’s (28) examined a cohort from developed urban areas in northern China, primarily focusing on individuals with metabolic syndrome. Lian et al. (27), on the other hand, recruited patients from western China, including some with pre-existing MI. In contrast, our study specifically targeted patients with CAD in southwestern China and employed a longer follow-up period. Differences in comorbid conditions among study populations may also contribute to variability in results. Wang et al. (32) reported findings similar to ours, noting that their study participants were more likely to have pre-existing cardiovascular conditions such as heart failure, hypertension, and atherosclerosis. Additionally, Daghlas et al. (31) conducted analyses at the genetic level, which were not included in our study.

### Sleep duration with CAD caused death

The association between sleep habits and CAD has been extensively studied, with evidence suggesting that both insufficient sleep (27) and excessive sleep (32, 33) may elevate the risk of CAD. For instance, Lian et al. (27) identified a strong correlation between sleep deprivation and CAD severity, while Cui et al. (33) reported that prolonged sleep duration was associated with an increased incidence of CAD in individuals over the age of 50. However, research specifically examining the relationship between sleep duration and CAD-related mortality within the Chinese population remains limited. Wang et al. (32) found that both short and long sleep durations were potential predictors of all-cause mortality. Similarly, our study observed that both insufficient and excessive sleep were linked to an increased risk of CAD mortality.

Our findings are largely consistent with those of Wang et al. (32), who also reported a heightened risk of CAD mortality in individuals with extreme sleep durations. However, while Wang et al.’s study reported HRs for all-cause mortality of 1.29 (95% CI: 1.08–1.55) for <6 hours of sleep and 1.77 (95% CI: 1.31–2.38) for >8 hours, our study found markedly higher HRs for CAD-specific mortality of 5.20 (95% CI: 2.53–10.68) for sleep duration of less than 6 hours, and 5.02 (95% CI: 1.59–15.80) for more than 8 hours. This suggests that the impact of short sleep duration on CAD-related mortality may be even more pronounced in our study population, aligning with previous findings (17, 34).

Several factors may contribute to these discrepancies. First, the two studies examined different mortality outcomes—our study focused specifically on CAD-related mortality, whereas Wang et al. (32) analyzed all-cause mortality. Additionally, our study population was generally older by approximately 10 years compared to that of Wang et al.’s (32). Furthermore, individuals with shorter sleep duration in our cohort were more likely to have lower educational levels and a higher prevalence of hypertension, DM, and hyperlipidemia, all of which are established risk factors for CAD mortality.

While previous studies have reported that longer sleep duration is more strongly associated with cardiac-related deaths (9, 35), it is important to consider that many individuals with prolonged sleep duration also have underlying health conditions that may contribute to increased mortality risk (9).

### Possible mechanism behind

The precise pathophysiological mechanisms linking sleep duration with MI and CAD-related mortality remain incompletely understood. However, several hypotheses and mechanisms have been proposed.

Empirical evidence suggests that both short and long sleep durations are associated with elevated levels of inflammatory markers, such as C-reactive protein (CRP) and interleukin-6 (IL-6), which contribute to endothelial dysfunction and arterial plaque formation (36, 37).

However, the mechanisms underlying short and long sleep duration may differ. Short sleep duration has been linked to increased coronary artery calcification and disturbances in circadian rhythms (36). Additionally, insufficient sleep disrupts endocrine and metabolic functions, leading to impaired glucose tolerance, insulin resistance (38) and decreased levels of key hormones such as leptin (9), testosterone, and melatonin (36). These metabolic disruptions are closely associated with the development of DM, atherosclerosis (38), and increased mortality (39). Furthermore, sleep deprivation has been shown to heighten sympathetic nervous system activity, resulting in elevated blood pressure and an increased likelihood of CAD events (38).

In contrast, prolonged sleep duration may not directly contribute to CAD but may instead serve as an indicator of underlying poor health, necessitating greater clinical attention to CAD risk in these individuals (9). Excessive sleep has been associated with poor sleep quality, frequent nocturnal awakenings, and autonomic nervous system imbalance, all of which contribute to cardiovascular stress (5). Additionally, studies have found that long-sleepers are at an increased risk of developing atherosclerosis (40). Extended periods of bed rest may also impair circulation and promote clot formation, particularly among elderly individuals with increased blood viscosity (41). Furthermore, reduced physical activity in long sleepers may exacerbate metabolic dysfunction, further elevating cardiovascular risk. Long sleep duration has also been linked to a range of adverse outcomes, including fatigue, DM, chronic inflammation, obstructive sleep apnea, depression, unemployment, and lower socioeconomic status (5, 42, 43). These factors collectively contribute to an increased burden of comorbidities and higher mortality rates.

Although sleep duration is an important predictor of CAD risk, it is influenced by multiple confounding factors, including socioeconomic status, depression, sedentary behavior, social isolation, and lifestyle habits. For example, individuals with lower socioeconomic status may have limited access to healthcare services, which can affect CAD diagnosis and treatment. Additionally, depression is closely associated with disrupted sleep patterns and has been independently linked to increased cardiovascular risk (44). Prolonged sedentary behavior, which often coexists with long sleep duration, may further contribute to metabolic dysregulation and CAD progression.

Notably, in our study, patients with short sleep duration tended to be older than those in the other groups. Prior research has suggested that short sleep duration is associated with poorer sleep quality, particularly among elderly individuals (45). Furthermore, lower educational levels may indicate a lack of awareness regarding healthy lifestyle choices. A higher prevalence of family history of CVD may also predispose these individuals to CAD.

Dietary habits are another important consideration. Our findings suggest that individuals with shorter sleep durations were more likely to consume a high-salt diet while having lower intakes of fruits and yogurt. This dietary pattern may be influenced by regional dietary preferences, as people in Southwest China traditionally consume high-salt and high-fat foods, such as pickled vegetables. On the other hand, fruits, vegetables, and yogurt are considered protective factors against CAD. High consumption of antioxidant- and fiber-rich fruits and vegetables has been shown to reduce systemic inflammation, lower low-density lipoprotein cholesterol, increase high-density lipoprotein cholesterol, regulate blood pressure, and modulate gut microbiota (46–48). Additionally, yogurt, which is rich in bioactive peptides and lactobacilli, has been found to reduce inflammation and improve lipid metabolism (49). Therefore, consuming a diet rich in these foods may help mitigate the adverse cardiovascular effects associated with extreme sleep durations (50). Future research should consider incorporating these dietary factors to further refine the understanding of the sleep-CAD relationship.

### Clinical significance

Our findings suggest that patients with CAD should aim for a sleep duration of 6–8 hours per day, as both insufficient and excessive sleep durations are associated with increased mortality risk. This insight highlights the importance of sleep as a modifiable lifestyle factor in CAD management. Encouraging CAD patients to maintain a healthy sleep duration may contribute to better cardiovascular outcomes, improved quality of life, and enhanced overall prognosis. Healthcare providers should integrate sleep assessments into routine clinical evaluations and offer targeted lifestyle interventions to promote optimal sleep patterns.

### Limitations

Despite the valuable insights gained from our study, several limitations must be acknowledged:

1. The sample size of CAD patients in our study is relatively small, and all participants were recruited exclusively from Southwest China. This limits the generalizability of our findings to broader populations. Future large-scale, multi-center studies are needed to confirm these results and enhance external validity.
2. This study employed a retrospective design, which inherently poses limitations compared to prospective studies. Although we performed an initial univariate adjustment and then conducted multivariate analysis, the presence of residual confounding factors cannot be ruled out, potentially introducing bias.
3. While baseline characteristics, comorbidities, and medication use were generally comparable across groups, the proportion of patients with sleep durations >8 hours was only 6.6% (n=54). This small subgroup size may introduce selection bias and limit the robustness of conclusions regarding long sleep duration.
4. Our study focused exclusively on self-reported sleep duration and did not assess other important dimensions of sleep, such as sleep quality, fragmentation, timing (e.g., bedtime and wake-up time), or daytime functional recovery. Although self-reported sleep duration is commonly used in epidemiological studies due to its practicality, it is subject to recall bias and may lead to over- or underestimation. Additionally, we did not account for factors like sleep onset latency, continuity, depth, and subjective sleep experience, which are known to influence both sleep quality and cardiovascular risk. These aspects can be more accurately evaluated through objective measures such as actigraphy or polysomnography (51, 52). Future research should integrate both subjective and objective tools to improve the reliability of sleep assessment and clarify the relationship between sleep and CAD outcomes.

## Conclusions

Our study demonstrates that both short (<6h/day) and long (>8h/day) sleep durations are associated with an increased risk of cardiac mortality among CAD patients in Southwest China. Notably, short sleep duration is also linked to a higher risk of MI, while long sleep duration does not show a significant association with MI.

## Data Availability

The original contributions presented in the study are included in the article/**Supplementary Material**. Further inquiries can be directed to the corresponding author.
