# Supplementary figures and images for: The influence of sleep duration on patients with coronary artery disease: a four-year observational study
Source: Front Endocrinol (Lausanne). 2025 May 5;16:1555880. doi: 10.3389/fendo.2025.1555880 (PMC12086895; doi:10.3389/fendo.2025.1555880)

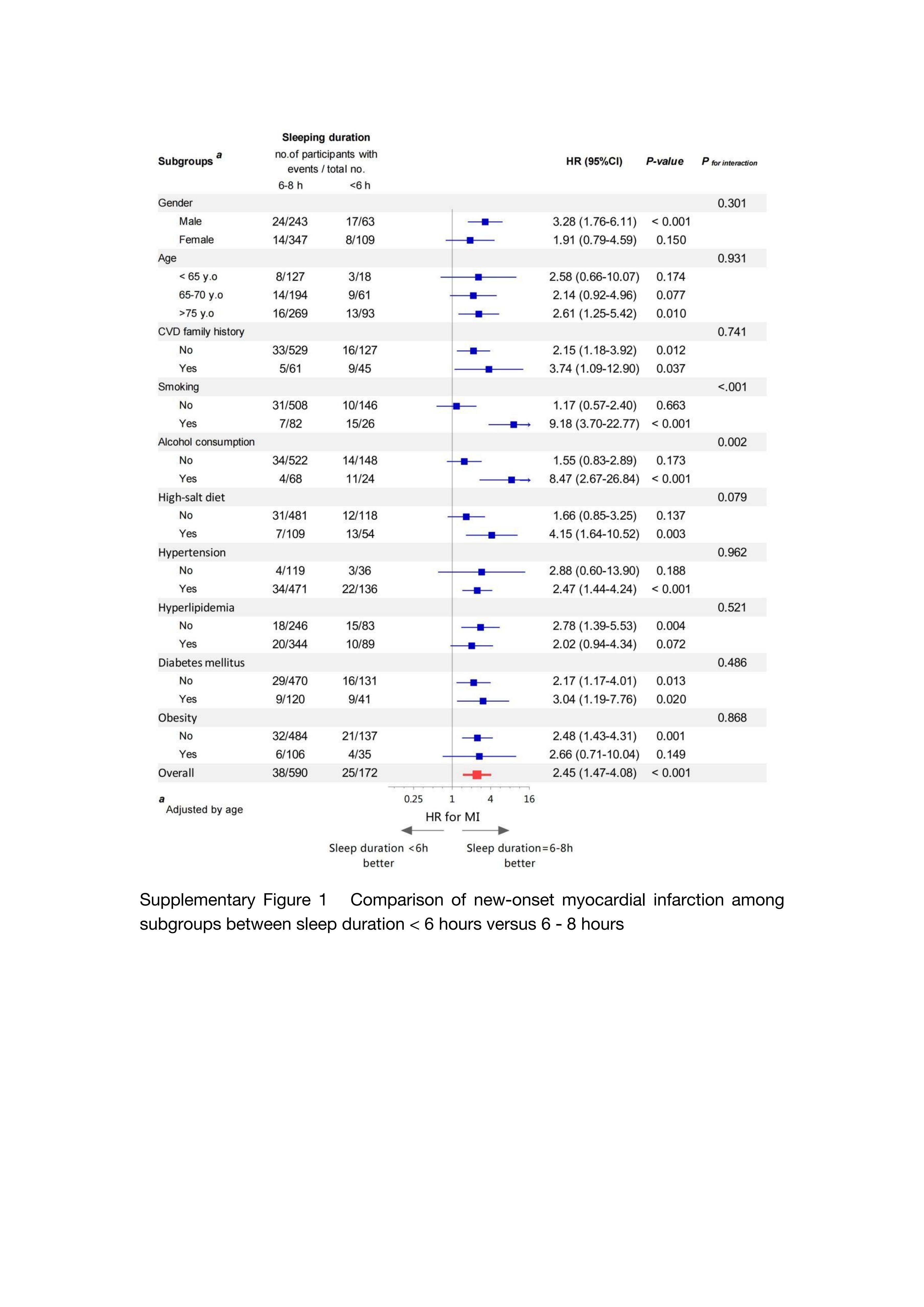

Supplement: Supplementary file 1 [file Image1.jpeg]

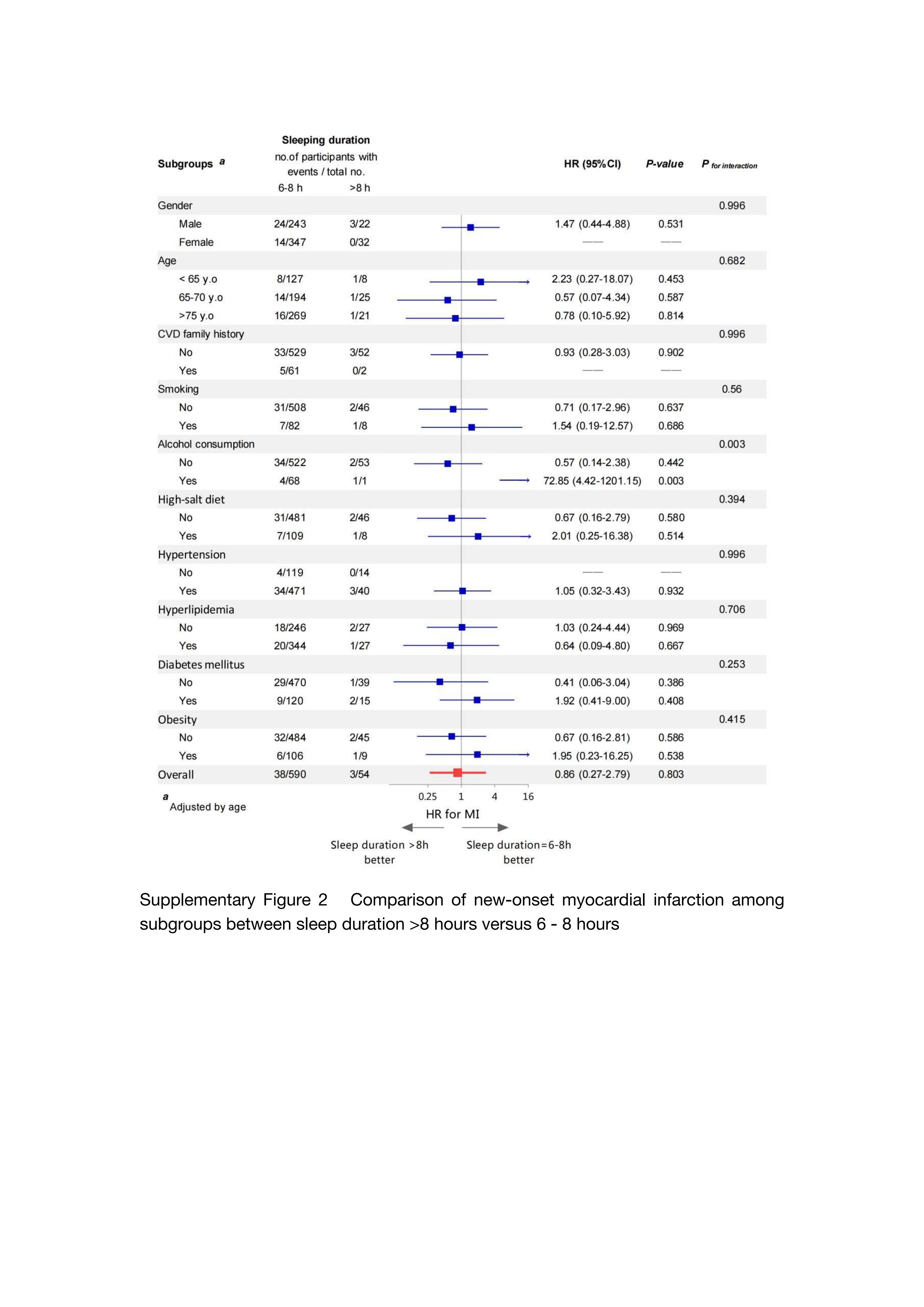

Supplement: Supplementary file 2 [file Image2.jpeg]

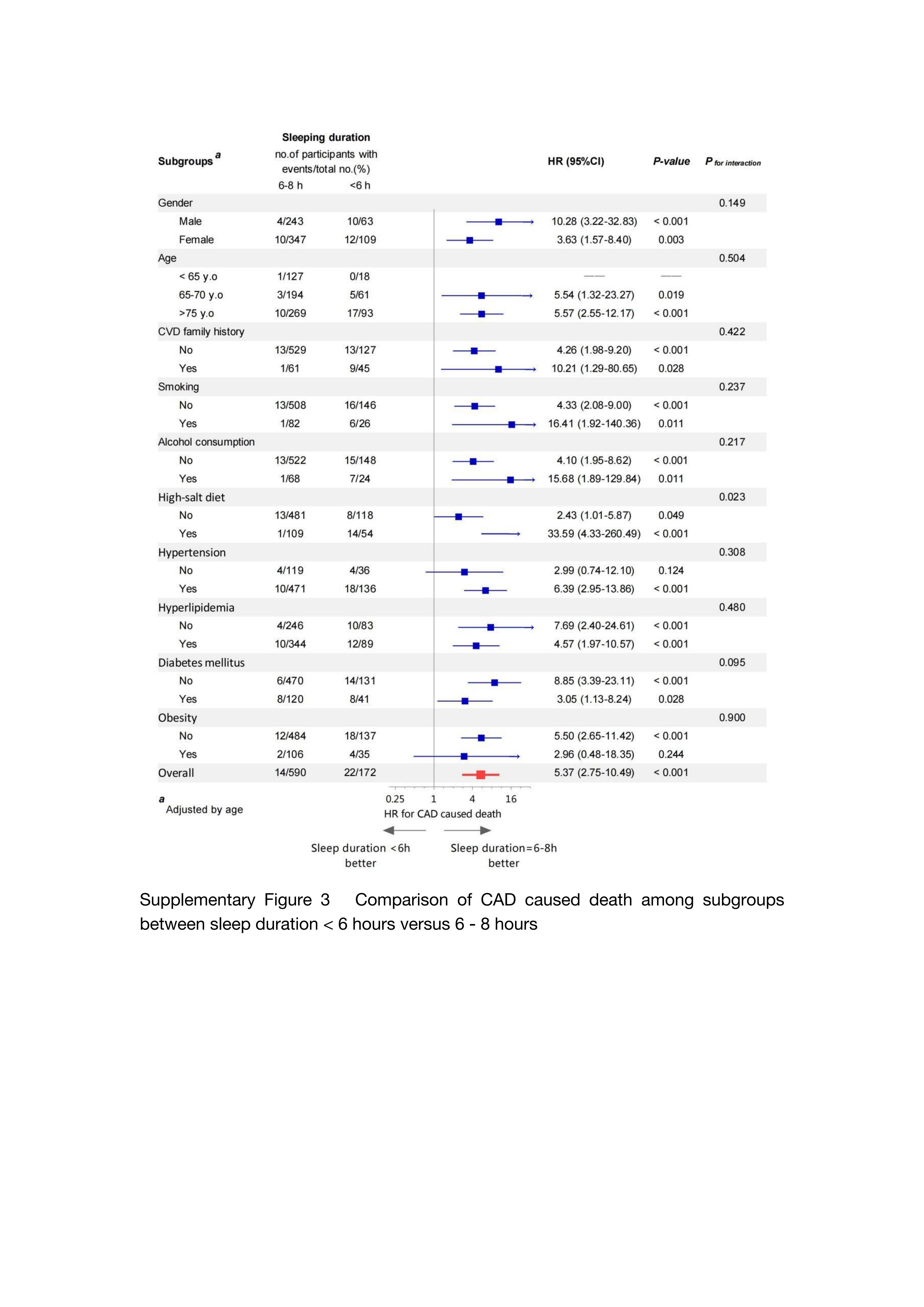

Supplement: Supplementary file 3 [file Image3.jpeg]

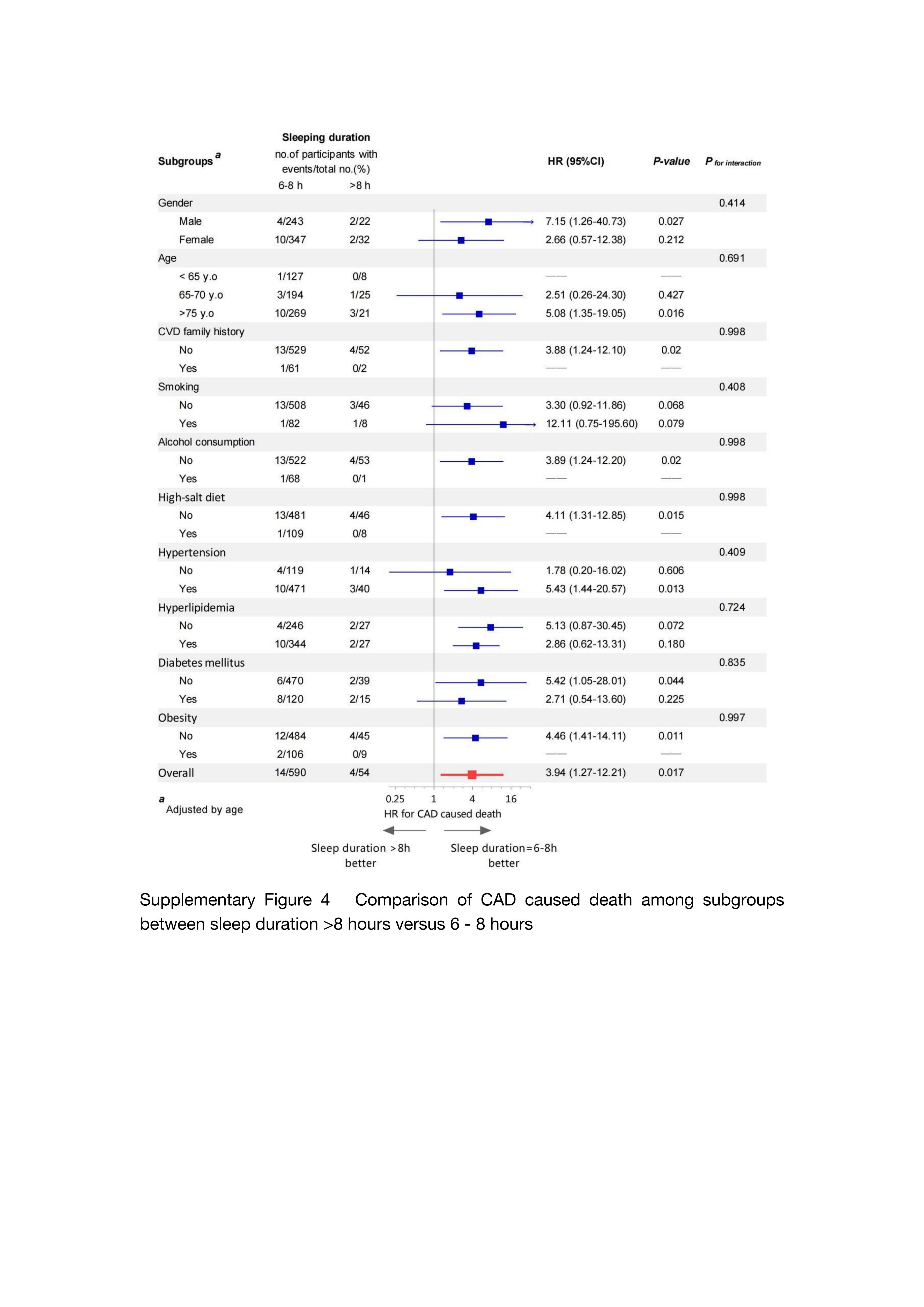

Supplement: Supplementary file 4 [file Image4.jpeg]
