# Supplementary material for: The influence of sleep duration on patients with coronary artery disease: a four-year observational study
Source: Front Endocrinol (Lausanne). 2025 May 5;16:1555880. doi: 10.3389/fendo.2025.1555880 (PMC12086895; doi:10.3389/fendo.2025.1555880)
Supplement: Supplementary file 5 [file Table1.pdf]

Supplementary Table 1 Univariate and multivariate Cox analysis for new myocardial infraction

Variable	Univariate analysis		Multivariate analysis [a]	
	HR (95% CI)	*P-value*	HR (95% CI)	*P-value*
Age [b]	1.01 (0.98-1.04)	0.677	1.01 (0.98-1.04)	0.592
Female gender	0.33 (0.20-0.54)	< 0.001	0.43 (0.23-0.78)	0.006
CVD family history	1.84 (1.02-3.32)	0.043	1.14 (0.60-2.18)	0.691
Smoking Alcohol consumption	3.49 (2.10-5.79)	< 0.001	2.10 (1.11-3.97)	0.023
	2.69 (1.53-4.72)	< 0.001	1.21 (0.62-2.34)	0.575
Physical inactivity	1.61 (0.99-2.62)	0.057	Excluded 1.49	
High-salt diet	1.86 (1.11-3.12)	0.019	(0.86-2.59)	0.155
Vegetables diet	0.74 (0.23-2.35)	0.607	Excluded 2.40	
Hypertension	2.24 (1.02-4.90)	0.044	(1.09-5.28)	0.029
Hyperlipidemia	0.67 (0.42-1.09)	0.110	Excluded	
Diabetes mellitus	1.66 (0.98-2.81)	0.057	Excluded	
Obesity	0.91 (0.48-1.75)	0.787	Excluded	
Anti-PLT	1.35 (0.69 2.64)	0.383	Excluded	
Lipid-lowering drugs Sleep duration	1.40 (0.78-2.52)	0.264	Excluded [c]	
6-8h	1.00		1.00	
< 6h	2.46 (1.48-4.07)	< 0.001	2.63 (1.54-4.49)	< 0.001
> 8h	0.86 (0.27-2.79)	0.803	0.98 (0.30-3.22)	0.975
7h	1.00		1.00	
≤ 5h	5.32 (2.16-13.12)	< 0.001	4.76 (1.84-12.32)	0.001
6h	0.92 (0.26-3.24)	0.892	0.85 (0.24-3.03)	0.800
8h	0.84 (0.24-2.99)	0.794	0.87 (0.24-3.08)	0.824
≥ 9h	2.97 (0.84-10.52)	0.092	4.45 (1.21-16.28)	0.024

Abbreviations: HR hazard ratio, CI confidence interval, CVD cardiovascular disease, PLT platelet

[a] Multivariate analysis was adjusted by age, female gender, CVD family history, smoking, alcohol consumption, high-salt diet, hypertension and sleep duration

[b] Age served as continuous variables

[c] Excluded as a confounder of hyperlipidemia

Supplementary Table 2 Univariate and multivariate Cox analysis for CAD caused mortality

Variable	Univariate analysis		Multivariate analysis [a]	
	HR (95% CI)	*P-value*	HR (95% CI)	*P-value*
Age [b]	1.12 (1.07-1.17)	< 0.001	1.15 (1.09-1.21)	< 0.001
Female gender	1.01 (0.54-1.90)	0.980	0.81 (0.42-1.53)	0.511
CVD family history	2.25 (1.10-4.61)	0.026	0.92 (0.41-2.03)	0.831
Smoking Alcohol	1.53 (0.71-3.32)	0.281	Excluded	
consumption				
	1.99 (0.92-4.33)	0.081	Excluded	
Physical inactivity	1.45 (0.77-2.72)	0.253	Excluded	
High-salt diet	2.34 (1.23-4.44)	0.009	3.11 (1.55-6.24)	0.001
Vegetables diet	0.31 (0.11-0.87)	0.027	0.10 (0.03-0.30)	< 0.001
Hypertension	0.91 (0.43-1.91)	0.801	Excluded	
Hyperlipidemia	1.16 (0.61-2.18)	0.653	Excluded	
Diabetes mellitus	3.03 (1.62-5.64)	< 0.001	3.19 (1.67-6.09)	< 0.001
Obesity	0.78 (0.33-1.87)	0.585	Excluded	
Anti-PLT	0.81 (0.29-2.28)	0.691	Excluded	
Lipid-lowering drugs	1.46 (0.69-3.06)	0.319	Excluded [c]	
Sleep duration				
6-8h	1.00		1.00	
< 6h	5.74 (2.94-11.22)	< 0.001	5.22 (2.55-10.72)	< 0.001
> 8h	3.20 (1.05-9.73)	0.040	4.87 (1.54-15.35)	0.007
7h	1.00		1.00	
≤ 5h	5.32 (2.16-13.12)	< 0.001	4.76 (1.84-12.32)	0.001
6h	0.92 (0.26-3.24)	0.892	0.85 (0.24-3.03)	0.800
8h	0.84 (0.24-2.99)	0.794	0.87 (0.24-3.08)	0.824
≥ 9h	2.97 (0.84-10.52)	0.092	4.45 (1.21-16.28)	0.024

Abbreviations: HR hazard ratio, CI confidence interval, CVD cardiovascular disease, PLT platelet

[a] Multivariate analysis was adjusted by age, female gender, CVD family history, high-salt diet, vegetables diet, diabetes mellitus, and sleep duration

[b] Age served as continuous variables

[c] Excluded as a confounder of hyperlipidemia
